# Are Hazard Assessment Methods in the Assessment of Chemical Alternatives Suitable for REACH?

**DOI:** 10.1021/acs.est.4c03979

**Published:** 2024-10-09

**Authors:** Rachel L. London, Juliane Glüge, Martin Scheringer

**Affiliations:** Institute of Biogeochemistry and Pollutant Dynamics, ETH Zürich, 8092 Zürich, Switzerland

**Keywords:** assessment of alternatives, GreenScreen, hazard assessment, regrettable substitution, multiple-criteria decision analysis (MCDA), PFASs

## Abstract

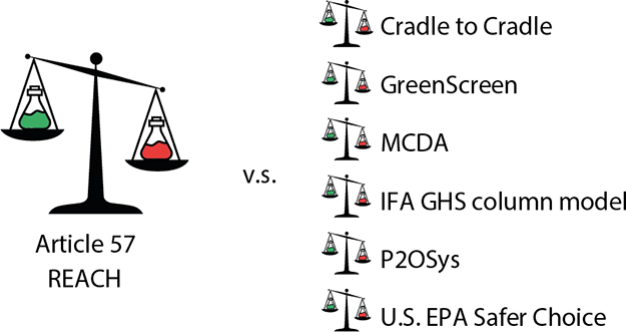

The assessment of chemical alternatives for hazardous substances is an important prerequisite for avoiding regrettable substitution, and several methods have been developed in the past to perform such a hazard assessment for chemical alternatives. We investigate here whether GreenScreen, Cradle to Cradle, multiple-criteria decision analysis (MCDA), the Pollution Prevention Options Analysis System, the U.S. EPA Safer Choice Standard and Criteria, and the GHS column model 2020 from IFA use similar criteria for the evaluation of substances as Article 57 of the European chemicals regulation, REACH, and how suitable these methods are for assessing per- and polyfluoroalkyl substances. MCDA and GreenScreen were analyzed in detail using two different data sets. The results of the assessments show that none of the investigated hazard assessment methods use the same criteria as described in Article 57 of REACH. It was also not possible to parametrize multi-attribute value theory (MAVT), a commonly used MCDA method, to align with Article 57 of REACH by using the relatively simple objective hierarchy that has been proposed in previous publications. There is therefore an urgent need for a modified/new method that can be used in the future to assess organic substances that are used within the European Economic Area.

## Abstract 2

To avoid regrettable substitution, the assessment of chemical alternatives is crucial. Therefore, assessing the alignment of current hazard assessment methods within Assessment of Alternatives frameworks with regulatory priorities, is required.

## Introduction

In February 2023, the European Chemicals Agency (ECHA) published a proposal for the restriction of per- and polyfluoroalkyl substances (PFASs)^1^ under the EU regulation on Registration, Evaluation, Authorisation, and Restriction of Chemicals (REACH). PFASs are substances of particular concern as they are persistent and many of the studied PFASs show effects that are problematic to human health and the environment. The proposed restriction on PFASs is—in terms of the number of substances covered—one of the largest restrictions that has so far been submitted under REACH. The PFAS definition that is used in the restriction proposal is similar to the OECD definition.^2^ Both definitions include substances with fully fluorinated methyl (−CF_3_) and methylene (−CF_2_−) groups. However, the restriction proposal under REACH excludes some specific subclasses of PFASs.^1^ Other definitions of PFASs exist,^3,4^ but for the purpose of this paper we mean PFASs as defined by the restriction proposal. PFASs have unique properties^1^ and these properties have led to the use of PFASs in many different applications.^5^ Alternatives are now being introduced for a range of PFAS uses and functions.^1,6−9^ However, while several nonhazardous alternatives have been found for some consumer uses of PFASs, it can be more complex for industrial uses, where the alternatives may have a different hazard profile, rather than being completely nonhazardous.^6^ For these alternatives, it is important to evaluate whether they represent a real improvement or whether they just replace one hazardous substance with another (regrettable substitution).

An example of regrettable substitution of PFASs was when perfluorooctanoic acid was replaced as a processing aid by 2,3,3,3-tetrafluoro-2-(heptafluoropropoxy)propionic acid (HFPO–DA) (the ammonium salt of which is sometimes referred to as “GenX”). In 2019, the EU also classified HFPO–DA as a Substance of Very High Concern (SVHC).^10^ By transitioning to a less regulated PFASs, rather than a less hazardous alternative, the fluorochemical industry did not solve the problem at hand. Having to perform a search for an alternative twice is an inefficient use of time, money, and expertise. The problem the present article seeks to address is how to minimize regrettable substitutions in the upcoming EU restriction of PFASs, but also for other chemical substitutions in the future.

To evaluate alternatives to hazardous substances and, if necessary, compare them with each other, Assessment of Alternative (AoA) frameworks have been developed.^11^ AoA frameworks consist in general of five sections: hazard assessment, exposure characterization, life cycle impacts, technical feasibility evaluation, and economic feasibility assessment.^11^ Performance in these sections is then aggregated by means of a decision-making logic specific to that framework. During the hazard assessment, physical, chemical, and toxicological hazards of the proposed alternatives are evaluated.

To minimize regrettable substitution, all relevant hazards should be considered, and a suitable hazard assessment method applied. For PFASs, relevant hazards include persistence (P), bioaccumulation (B), ecotoxicity (T_eco_), human toxicity (T_hu_), mobility (M), global warming potential (GWP) and ozone depletion potential (ODP).^1^ These hazards are regulated in Europe in different ordinances. P, B, T_eco_ and T_hu_ are included in REACH (specifically in Article 57 of REACH),^12^ while GWP is addressed in the European F-Gas Regulation.^13^ GWP and ODP are globally regulated in the Montreal Protocol and its Kigali Amendment.^14^ Mobility has so far only been included as a specific criterion in the Classification, Labeling and Packaging (CLP) Regulation of the EU.^15^ The present article focuses specifically on Article 57 of REACH and investigates: (1) if current hazard assessment methods that are used within AoA frameworks align with Article 57 of REACH, and (2) if these hazard assessment methods are appropriate for PFASs as a particular group of chemicals of concern.

To do so, the paper investigates various available hazard assessment methods, including GreenScreen,^16^ Cradle to Cradle,^17^ the Pollution Prevention Options Analysis System (P2OSys),^18^ the U.S. EPA Safer Choice Standard and Criteria,^19^ and the Institute for Occupational Safety and Health of the German Social Accident Insurance’s (IFA’s) GHS column model 2020 (ref (20)) for their suitability for assessing chemicals in the same way as Article 57 under REACH. Additionally it was investigated how multiple-criteria decision analysis (MCDA) was used in previous publications to perform a hazard assessment and if the setup of these methods aligns with Article 57 of REACH.

Article 57 of REACH is part of the identification of SVHCs.^12^ SVHCs may be included in Annex XIV of REACH, which is the list of substances subject to authorization. The complete text of Article 57 is given elsewhere,^12^ but in short, it addresses substances that are carcinogenic, mutagenic, or toxic for reproduction (CMR), or persistent, bioaccumulative and toxic (PBT) or very persistent and very bioaccumulative (vPvB) or endocrine disruptive or of “equivalent concern”. If a substance has been officially identified under REACH as an SVHC, it will be added to the so-called Candidate List of SVHCs for authorization.

## Methods

### Overview of Hazard Assessment Methods

The methods that were investigated in the present study were identified by reviewing the AoA frameworks in Jacobs et al.^11^ for their hazard assessment methods. The hazard assessment section of AoA frameworks is of interest to this paper, because this is the AoA section relevant to Article 57 of REACH. From this, the following methods were identified: Cradle to Cradle,^17^ GreenScreen,^16^ the GHS column method 2020 from IFA,^20^ P2OSys,^18^ and the US EPA Safer Choice Standard and Criteria.^19^ For GreenScreen, the criteria of the Hazard Assessment Guidance^16^ were investigated but not the GreenScreen certification standards for the seven product classes.^21−27^

In addition to these methods, MCDA was also selected to be reviewed.^28^ MCDA was defined as a decision making tool by Jacobs et al.,^11^ but MCDA has also been used for the hazard assessment of chemical alternatives in the recent literature^29−33^ and in policy documents.^34,35^

For each of the hazard assessment methods, it was investigated which hazards are covered by the methods, which thresholds are used to classify the hazards and how the hazards are aggregated. Also, it was investigated whether the hazard assessment method covers all hazards relevant to PFASs (P, B, T_eco_, T_hu_, M, GWP, ODP), how the methods treat organohalogens, if there are any PFAS-specific rules and if degradation products are covered.

In order to select two methods for a more detailed analysis, the methods were first categorized according to their decision logic. A distinction was made between decision trees (GreenScreen, Cradle to Cradle, GHS column method 2020 from IFA, and US EPA Safer Choice Standard and Criteria) and weighted sums (MCDA and P2OSys). One method was then selected from each group. GreenScreen was selected for the decision trees, because the criteria in GreenScreen are very similar to the ones in Article 57 of REACH, because GreenScreen has been recommended in ECHA training,^36^ and because it is also possible with GreenScreen to differentiate between imperfect alternatives. These could be alternatives were there are mild to moderate concerns about one or several endpoints. This contrasts to Cradle to Cradle or US EPA design for environment, whose purpose is to identify exceptionally low-hazard alternatives. MCDA was selected as a weighted sum as it is the more flexible method compared to P2OSys in terms of method parameters (e.g., aggregation).

### Data Sets Used to Investigate the Methods In-Depth

To be able to use data in the assessment of the two methods, five general data treatment steps are necessary: data gathering, data normalization, data conversion, data aggregation, and data classification. Data gathering means that relevant hazard data (e.g., the degradation half-life of a substance) need to be collected. Data normalization describes the step where the gathered data are categorized (normalized) into hazard levels using external thresholds. For example, a half-life can describe the persistence of a substance as low, moderate, or high. The allocation to the hazard levels depends in this case on the half-life itself, but also on the thresholds used. The third step is the conversion of the hazard levels into a quantitative hazard value. This is followed by the data aggregation step where the normalized hazard values of the different endpoints (persistence, bioaccumulation, etc.) are combined into one final score. The final step is the classification of the substance according to the final score. To investigate MCDA and GreenScreen in detail, two different data sets were used, a “hypothetical substances data set” and a “real substances data set”.

#### Hypothetical Substances Data Set

For the hypothetical substances data set, no actual data were gathered, instead 256 different combinations of four hazards and four hazard severities were used to investigate the conversion of the qualitative hazard data into hazard values as well as the data aggregation and data classification step in detail. The four hazards included were those relevant to both GreenScreen and Article 57 of REACH: persistence (P), bioaccumulation (B), human toxicity (T_hu_) and ecotoxicity (T_eco_). Mobility (M) was not considered explicitly for the hypothetical substances. The four hazard severities were based on those used within the GreenScreen method: very high, high, moderate, and low. By using hypothetical substances, it was possible to systematically investigate how the two methods, MCDA and GreenScreen, respond to each combination of hazard and hazard severity within the scope of the study.

#### Real Substances Data Set

The second data set used to investigate MCDA and GreenScreen in detail was a data set published in a previous article^30^ for the comparison of 17 substances (the brominated flame retardant decabromodiphenyl ether and 16 alternatives) using MCDA. The hazards considered in this data set were P, B, M and toxicity (which included both T_eco_ and T_hu_). Some of the data points had been determined experimentally; others were derived from quantitative structure–activity relationships (QSARs) alone. The data normalization was done by using the thresholds from the Guidance on Information Requirements and Chemical Safety Assessment Chapter R.11: PBT/vPvB assessment.^37,38^ The normalized data were then used to investigate the data aggregation and data classification in the two methods, MCDA and GreenScreen. Important to note here is that the data set of Zheng et al.^30^ did not include uncertainty ranges of the hazard data. However, as the primary purpose of the present article is an investigation into the methods, these data limitations were considered acceptable.

### Investigations into GreenScreen

**Figure 1:**
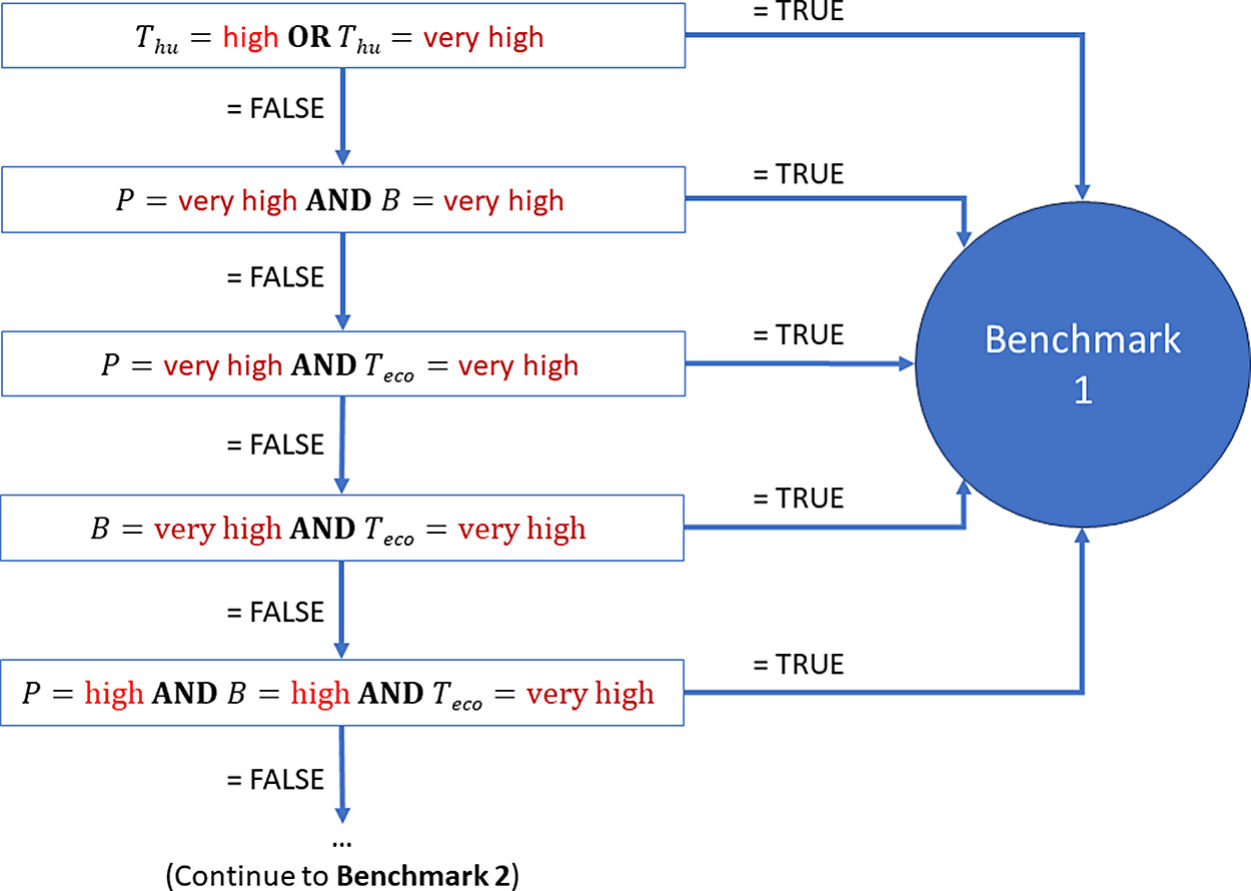
Section of the decision tree used to replicate GreenScreen (Benchmark 1) P = Persistence, B = Bioaccumulation, T_eco_ = Ecotoxicity, T_hu_ = Human Toxicity.

A spreadsheet (SI-2) was created to categorize all substances in both data sets according to the GreenScreen decision tree, as described in Annex 3 of the Guidance^16^ and shown in SI-1Figure S1. This was done by transforming the GreenScreen decision tree into AND, IF, and OR statements, as shown in Figure 1 for Benchmark 1 (“Avoid: chemical of high concern”).

If Benchmark 1 gave a FALSE for all decision points, the hazard scores were assessed using an equivalent process for Benchmark 2. This process was repeated for subsequent benchmarks, until the substance was categorized. Physical hazards were excluded from the assessment, as these data are not relevant for Article 57 of REACH. For the real substances data set, it should be noted that approximate assessments were made for the GreenScreen endpoints using solely the data already provided by Zheng et al.^30^ These data were not always exactly of the type or format required by the GreenScreen guidance (see SI-1 Section S2.1); however, they were sufficient for the investigation of the method. It is still important to note that the GreenScreen assessments made here should not be used outside the present article.

### Investigations into the Variability of MCDA

For MCDA, it was first investigated how different curvatures, aggregations and different weighting influence the outcome of the results. To this end, MCDA was performed on the two data sets, and the outcome then contrasted with the outcome of GreenScreen. The MCDA method chosen was multi-attribute value theory (MAVT). MAVT was chosen because it is a robust method which is known not to suffer from rank reversal (unlike other MCDA methods such as ELECTRE, AHP, PROMETHEE, and possibly DSA).^39^ Reichert et al.^40^ also advocated for the use of MAVT over other MCDA methods in environmental decision-making. In prior literature on MCDA used in assessments of chemical alternatives, no significant difference was found between the outcomes of MAVT and ELECTRE.^30,41^ Notably, there is a discrepancy in the method terminology between this paper, which uses “MAVT,” and the prior literature, which uses the term “MAUT.” The term MAVT should be used when the data evaluated do not include uncertainty (or are assumed to be certain).^42^

MAVT calculates a value, *f*, which is the MCDA hazard score in the present article. The MCDA hazard score can range from zero (worst) to one (best) for each alternative by aggregating the performance in objectives (e.g., low persistence) so that the alternatives can then be ranked from most to least desirable. The MCDA-MAVT method parameters that are most appropriate for an assessment of chemical alternatives had not yet been investigated in the literature. Therefore, to determine these parameters, a bounding analysis was done on different combinations of the parameters aggregation, curvature of the value function, and weighting of objectives.^43^ The objective hierarchy chosen was the one from Zheng et al.^30^

For the investigations, the MCDA tool, ValueDecisions,^43,44^ was used. Five different aggregations were investigated: additive (eq 1), geometric (eq 2), geometric-additive (eq 3), minimum (eq 4), and maximum aggregation (eq 5).
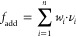
1
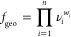
2
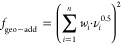
3
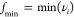
4
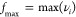
5

The equation for the five different aggregations were originally developed by Langhans et al.^45^ and are included in ValueDecisions. For each alternative considered, the MCDA hazard score, *f*, is calculated using one of the aggregation equations above. The subscript *i* denotes different objectives in the MCDA. The variable ν_*i*_ (0.0 ≤ ν_*i*_ ≤ 1.0) represents the alternative’s performance in terms of the corresponding objective (*i*). ν_*i*_ is determined by transforming and combining attributes (e.g., half-life in water) into a single representative value. This transformation is done using value functions, mathematical expressions that convert the attribute data into comparable values, the output of which is then combined into ν_*i*_. A weight of an objective, *w*_*i*_, is also required in some of the aggregation eqs (0 ≤ *w*_*i*_ ≤ 1 where ∑ *w*_*i*_ = 1).

For the value functions, three different curvatures were investigated: linear, exponential-concave, and exponential-convex. The respective equations are provided in the SI-1 Section S3.1. Based on these equations, the following values were used to create the different value functions. Concave curvature: 0.456 (very high), 0.848 (high), 0.961 (moderate), 0.994 (low); linear curvature: 0.12 (very high), 0.37 (high), 0.62 (moderate), 0.875 (low); convex curvature: 0.006 (very high), 0.036 (high), 0.144 (moderate), and 0.532 (low). For the weights, three different weights (*w*_P_) were investigated by varying the weight of the objective of persistence relative to the three other objectives: equal (*w*_P_ = 0.25), high (*w*_P_ = 0.55), or low (*w*_P_ = 0.15). These weights were chosen to ensure that they summed to one in each scenario, therefore for the equal weight *w*_P_ = 1/4 = 0.25. Marttunen et al.^46^ cautioned against utilizing “very low weights” (≤0.05); thus, the low weight was established equidistant from 0.05 and 0.25 at 0.15. The high weight was derived once the low weight had been established. If all four weights must sum to one, and there are three “low” weights of 0.15, the remaining single “high” weight must be 0.55 (*w*_P_ = 1–3·0.15 = 0.55).

**Figure 2:**
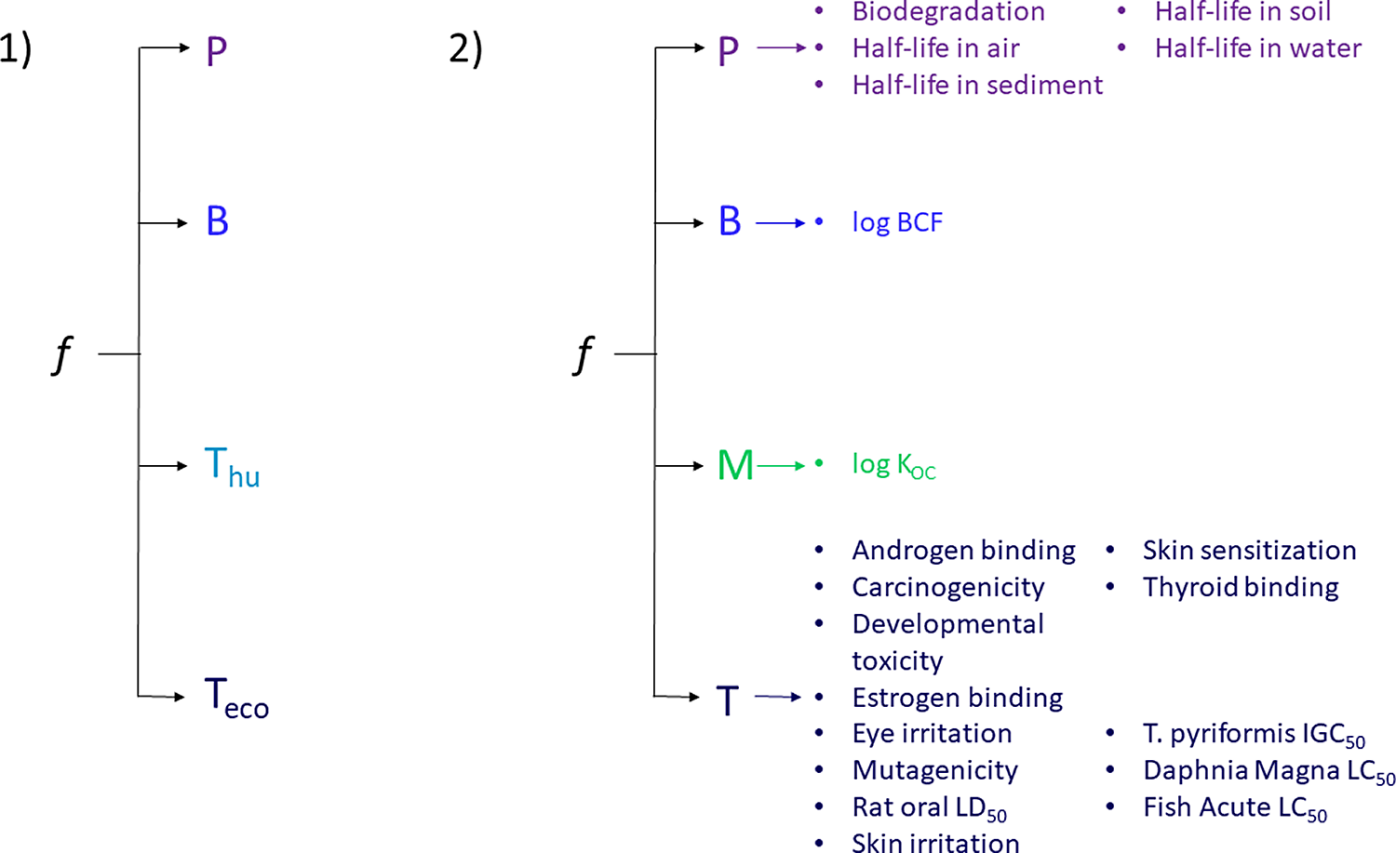
MCDA objective hierarchy showing the hazards that contribute to the MCDA hazard score (*f*) of a substance for (1) the hypothetical substances data set and (2) the real substances data set. *P* = persistence, B = bioaccumulation, M = mobility, *T* = toxicity, T_eco_ = ecotoxicity, T_hu_ = human toxicity.

From the hypothetical substances data set, a subset of 17 substances with low (4), medium (7), and high variation (6) in hazard severity were selected (SI-2). Substance 86 is an example of a substance with low variation of hazard severity (all hazards = “high”), while Substance 64 is an example of a substance with high variation of hazard severity (persistence = “very high,” all other hazards = “low”). In the first investigation using the hypothetical data set, 15 MCDAs were conducted each with a unique parameter combination of the five different aggregations and the three different curvatures, while the weights were kept constant (equal, *w*_*i*_ = 0.25). In the second experiment of the hypothetical data set, 15 MCDAs were conducted each with a unique parameter combination of the five different aggregations and the three different weights, while the curvature was kept constant (linear). For the real substance’s data set, also 15 MCDAs were conducted with aggregation and curvature varied, while weights kept the same (equal, *w*_*i*_ = 0.25). The objective hierarchy used for each of these data sets is shown in Figure 2.

### Selecting MCDA Method Parameters to Align with Article 57 of REACH

The ability of MCDA to classify substances as potential SVHCs was investigated by using the hypothetical substances data set. In a first step, all hypothetical substances were manually assigned a hazard category, which was either “potential SVHC” or “not an SVHC” according to the criteria laid down in Article 57 of REACH.

In a second step, nine different MCDAs, each with a different set of parameters, were applied to the hypothetical substances data set. The parameters were a combination of three different aggregations (additive, geometric mean, and minimum) with three different curvatures (linear, exponential-convex, and exponential-concave) with equal weights throughout (*w*_*i*_ = 0.25) (SI-2). The discrete values used for the three curvatures are the same as in the previous section.

Finally, a classification threshold for *f* was defined below which a substance would be classified as equivalent to a “potential SVHC.” For all nine MCDA parameter combinations described above, the classification threshold for *f* was optimized so that the MCDA outcome had the highest agreement with the evaluation according to Article 57 of REACH. This was done by initially setting the classification threshold to 0.1 and increasing it incrementally from 0.1 to 0.9. Subsequently, the range of *f* was determined in which the highest match occurred. However, only one explicit threshold value for the MCDA outcome, *f*, was selected for the results section.

## Results

### Overview of Hazard Assessment Methods

The evaluation of the six hazard assessment methods is summarized in Table 1. Detailed descriptions of the methods and additional information are provided in SI-1 Section S1.1.

**Table 1:** Overview of Hazard Assessment Methods Investigated (Extended Version in SI-1, Section S1.2)

Hazard assessment method	Similar to the criteria in Article 57 of REACH?	How are PFASs treated?
**Cradle to Cradle**	Stricter than the criteria in Article 57. Failure in a single endpoint sufficient for exclusion. Therefore, persistence alone is sufficient to classify substance as unacceptable. Some endpoint thresholds (PURPLE and RED) are the same as those used in REACH for equivalent attributes (e.g., half-life in freshwater). See SI-1 Section S.1.3 for comparison of persistence and bioaccumulation thresholds.	Presence of organohalogen bonds renders a chemical unacceptable. All PFASs are therefore considered unacceptable under this method.
**GreenScreen** Hazard Assessment Guidance (2018)	Classifies some substances different than Article 57. Also, endpoint thresholds differ from those used in REACH. See SI-1 Section S1.3 for comparison of persistence and bioaccumulation thresholds.	Current method not appropriate for PFASs as it does not evaluate all relevant hazards of concern (does not cover mobility, GWP, and ODP). Additionally, PFASs classified as (only) vP would be classified as ‘3 = Use, but still opportunity for improvement’
**Multiple-criteria decision analysis (MCDA)**	Under the relatively simple objective hierarchy that has so far been applied in hazard assessments with MCDA, the output of the MCDA does not align with Article 57 of REACH. However, using a modified objective hierarchy might enable the alignment with Article 57 of REACH.	The method can potentially be applied to PFASs. It is imperative that suitable method parameters are selected.
**The GHS column model 2020 from IFA (the Institute for Occupational Safety and Health of the German Social Accident Insurance)**	Differences from REACH Article 57 include:	Endpoints covered do not currently capture all hazards of PFASs (e.g., mobility missing). Problematic degradation products are not addressed.
	• Endpoints covered (e.g., endocrine disruption not addressed)	
	• Severity that is assigned to different endpoints (e.g., toxicity to reproduction not equivalent to carcinogenicity or mutagenicity)	
	• How endpoints are aggregated (trade-offs between hazards possible)	
**MA TURI’s Pollution Prevention Options Analysis System (P2OSys)**	Environmental hazards are combined in a different way than in Article 57 of REACH. Thresholds differ from those used under REACH.	PFASs have a high variation of hazard severity. By taking an average of the category scores, this method allows for poor performance in one area (e.g., environmental fate) to be compensated for by good performance in another (e.g., acute human effects). The final scores given to substances with high variation of hazard severity are “good to average”. This means this method may be misleading if applied to PFASs.
**U.S. EPA Safer Choice Standard and Criteria**	Environmental endpoints (P, B, and T) are combined in both methods, but the implications of these combinations differ. US EPA penalizes additional hazard combinations (PT and P). Thresholds of P and B are stricter than those in REACH. Threshold of P varies depending on the acute aquatic toxicity. Additionally, assessment criteria can be modified for specific functional classes (e.g., surfactants, solvents).	Does not consider all PFAS-relevant hazards, e.g., mobility is missing. Persistent degradation products would exclude PFASs from being considered a “safer choice” substance. Proposed revisions to the guidance explicitly prohibit the intentional addition of PFASs to packaging.^47^

GreenScreen and MCDA were identified to be investigated further: the flexibility of MCDA made it an interesting method to see whether there is a parameter combination that allows the alignment of MCDA with Article 57 of REACH. GreenScreen was selected for the decision trees, as the criteria in GreenScreen are very similar to the ones in Article 57 of REACH, and it is also possible with GreenScreen to differentiate between imperfect alternatives.

The other methods were excluded from further investigation for various reasons. Cradle to Cradle and U.S. EPA Design for Environment are certification standards that can be used to identify consumer products with exceptionally low hazard. However, they have limited flexibility and may not be suitable where one must differentiate between imperfect alternatives, rather than identify a hazard-free one. The GHS column model 2020 from IFA is a decision-making system with no flexibility and a scope built around the current Globally Harmonized System of Classification, Labeling and Packaging of Chemicals (GHS) criteria. P2OSys does not align with Article 57 of REACH as the aggregation used in P2OSys allows poor performance in one hazard category to be compensated for by good performance in another hazard category.

### Investigations into GreenScreen

The hypothetical substances and the substances from the real substances data set were all categorized into one of four Benchmarks: 1 = “Avoid: chemical of high concern,” 2 = “Use but search for safer substitutes,” 3 = “Use but still opportunity for improvement,” 4 = “Prefer: Safer chemical.” For the real substances data set, GreenScreen categorized all substances into Benchmark 1. For the hypothetical substances data set, there were substances in all four groups. Table 2 displays how the 256 hypothetical substances were categorized by GreenScreen and compares this to their classification when the criteria from Article 57 of REACH are used. The comparison shows that eight substances were categorized as GreenScreen Benchmark 1 although they would not be considered as potential SVHCs under REACH. On the other hand, six substances are potential SVHCs under REACH, but received a GreenScreen Benchmark 2. Table 3 presents more detailed information for a subset of these substances. Detailed information for all other substances is available in the SI-2.

**Table 2:** Comparison of the Assessment of the Hypothetical Substances using GreenScreen and the Hazard Criteria Laid Down in Article 57 of REACH

GreenScreen Benchmark	Potential SVHC under REACH?	
	No	Yes
1 (“Avoid: chemical of high concern”)	8	142
2 (“Use but search for safer substitutes”)	77	6
3 (“Use but still opportunity for improvement”)	22	0
4 (“Prefer: Safer chemical”)	1	0

**Table 3:**
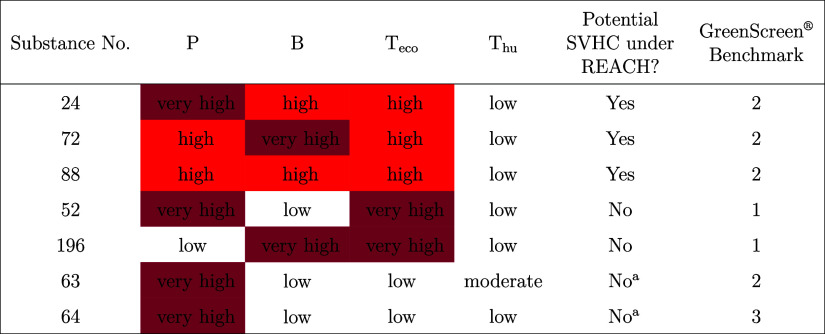
Selected examples of GreenScreen Benchmarks from the Hypothetical Dataset, Where 1 = “Avoid–Chemical of High Concern,” 2 = “Use but Search for Safer Substitutes,” 3 = “Use but Still Opportunity for Improvement,” 4 = “Prefer–Safer Chemical”

a If only the endpoints P, B, T_eco_, and T_hu_ are considered.

Table 3 shows that GreenScreen categorizes substances with a high or very high hazard for P, a high or very high hazard for B and a high hazard for T_eco_ as Benchmark 2 while these substances would be potential SVHCs under REACH. Substances with a hazard combination of very high P and very high T_eco_ or very high B and very high T_eco_ are otherwise Benchmark 1 in GreenScreen while these substances are not explicitly addressed in Article 57 of REACH. This means that GreenScreen is less strict than Article 57 of REACH with the categorization for some substances and stricter than Article 57 for others.

Substances 63 and 64 correspond to the hazard profile of PFASs, such as HFPO–DA, and were categorized as Benchmark 2 and 3. However, Benchmark 1 would have been more appropriate for HFPO–DA, as it has been classified as an SVHC.^10,48^ Even if the current criteria under Article 57 of REACH do not (yet) include mobility, substances that are very persistent and very mobile may be considered as substances with equivalent level of concern to CMR, PBT, and vPvB substances.^49^ In this respect, GreenScreen is less strict than Article 57 of REACH. To assess HFPO–DA and other PFASs with a similar hazard profile, the scope of GreenScreen would need to be expanded to include mobility, although mobility was not the only criteria that let to the decision to classify HFPO–DA as a SVHC.

### Dependency of the MCDA Hazard Scores on the Method Parameters

#### Hypothetical Substances Data Set

**Figure 3:**
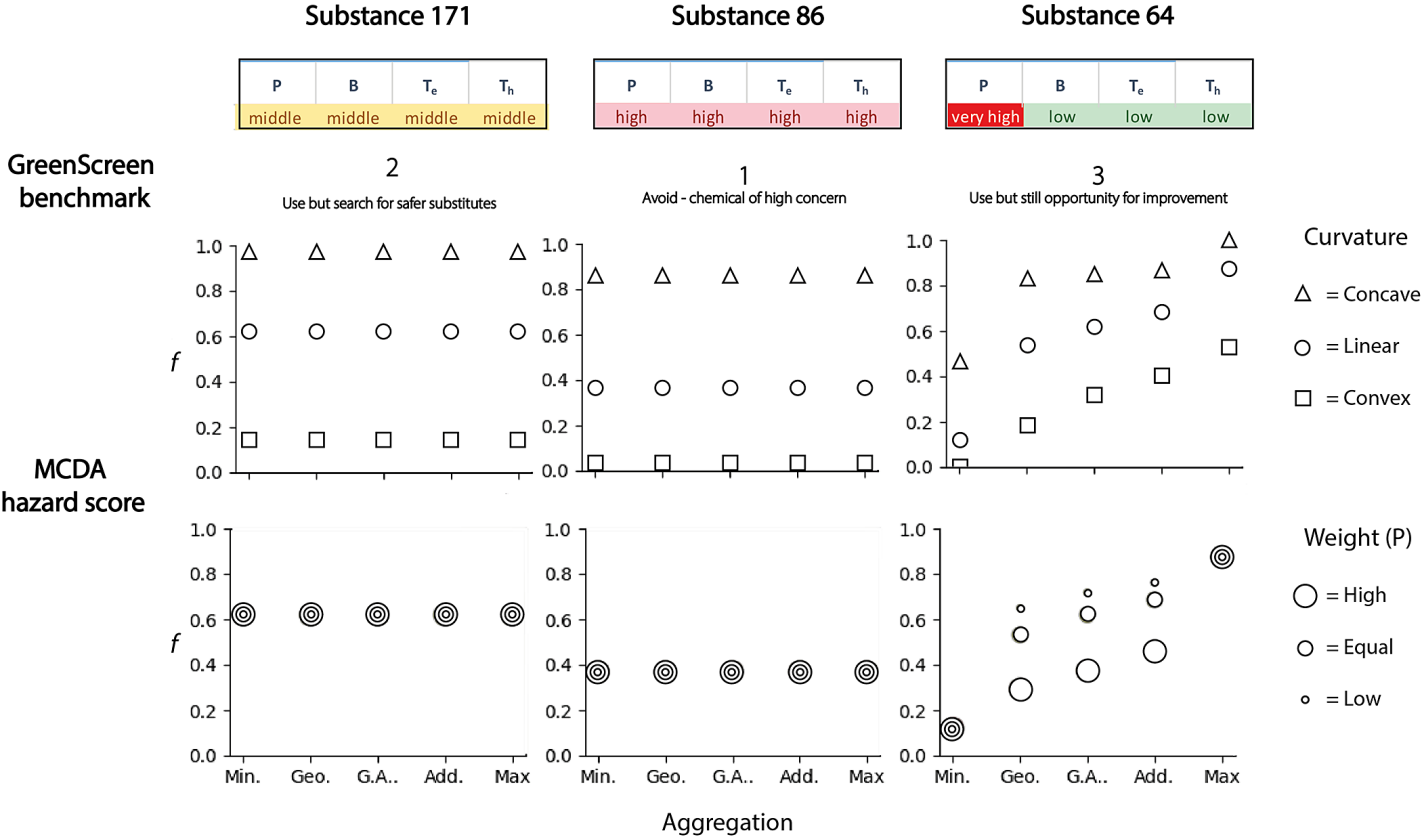
Variability of MCDA hazard scores (*f*) for three of the hypothetical substances (two with low variation of hazard severity, one with high variation of hazard severity). Top row: *f* as a function of curvature and aggregation, weights kept constant (equal, *w*_*i*_ = 0.25 for each hazard). Bottom row: *f* as a function of weights and aggregation, curvature kept constant (linear). (Min. = minimum aggregation, Add. = additive aggregation, Max. = maximum aggregation, Geo. = weighted geometric mean aggregation, G.A. = weighted geometric mean–additive aggregation).

To investigate the variability of MCDA outcomes, MCDA hazard scores (*f*) were determined for a subset of hypothetical substances, with 1 representing the best possible score and 0 the worst possible score. Figure 3 shows how *f* changes when the parameters of (1) aggregation and curvature and (2) aggregation and weighting are changed.

Substances 171 and 86 show that for substances with low variation of hazard severity (meaning all endpoints have the same hazard severity), *f* remains the same when aggregation and weight are varied, but changes in most cases when the curvature is varied (Figure 3). *f* is sensitive to changes in curvature because the three value functions—exponential-concave, linear and exponential-convex—only have the same value ν(*x*) at the beginning and end of the functions, where *x* represents the minimum or maximum value (e.g., the lowest and highest possible half-life values.) At all other points along the *x*-axis, the values of ν(*x*) diverge among the three functions. Figure S2 in SI-1 shows this again graphically, alongside a more detailed explanation.

Substance 64 shows that for a substance with high variation of hazard severity (meaning some hazards are very high and some are low), *f* is sensitive to the aggregation, curvature, and sometimes sensitive to the weighting. Whether *f* is sensitive to weights depends on the aggregation used because while weights influence the geometric, additive, and geometric-additive aggregation (eqs 1, 2, and 3), weights are not considered in minimum and maximum aggregation (eqs 4 and 5). This can also be seen in Figure S3 in the SI-1.

Substance 64 has the same hazard profile as some PFASs, such as HFPO–DA. So particular care should be taken in selecting the method parameters to be used for an MCDA involving PFASs, as they are often substances with a high variation of hazard severity, and so *f* determined for these substances will be sensitive to aggregation, curvature, and sometimes weights.

#### Real Substances Data Set

**Figure 4:**
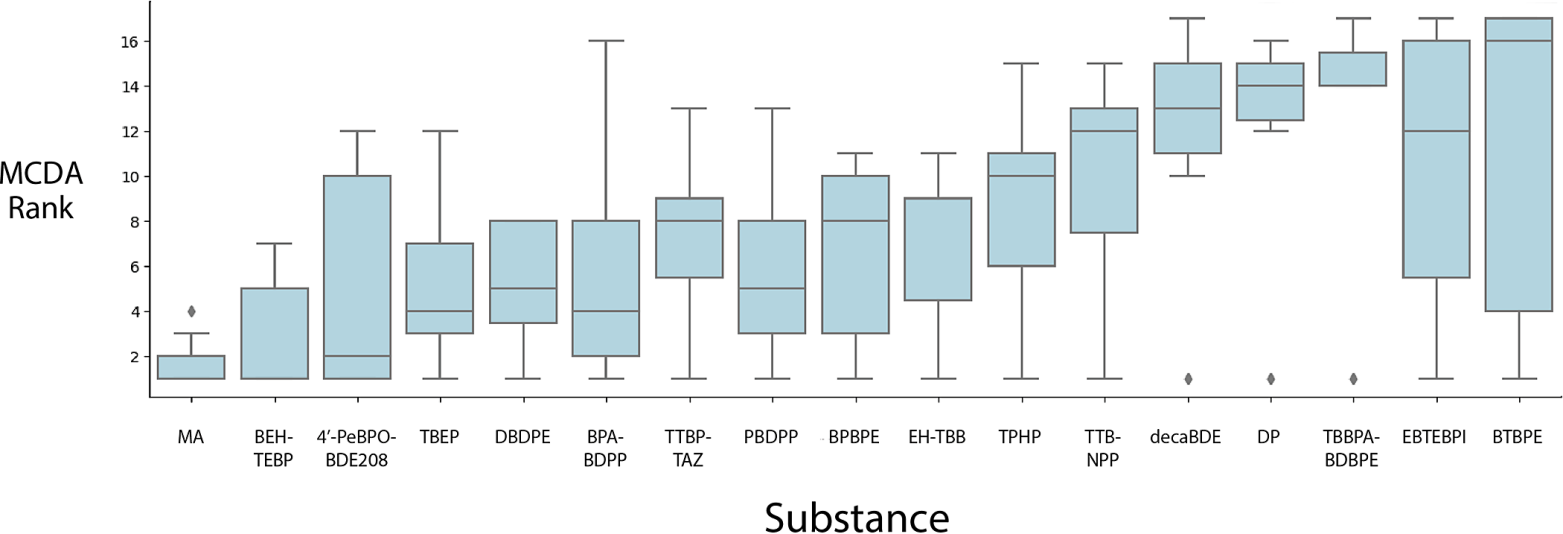
MCDA ranks for the real substances data set. Rank as a function of curvature and aggregation, weight kept constant. (MA = melamine, BEH-TEBP = bis(2-ethylhexyl) tetrabromophthalate, 4′-PeBPOBDE208 = tetradecabromodiphenoxybenzene, TBEP = tris(2-bromoethyl) phosphate, DBDPE = decabromodiphenyl ethane, BPA-BDPP = bisphenol A diphenyl phosphate, TTBP-TAZ = tris(tribromophenoxy) triazine, PBDPP = resorcinol bis(diphenyl phosphate), BPBPE = 1,2-bis(pentabromophenoxy) ethane, EH-TBB = 2-ethylhexyl tetrabromobenzoate, TPHP = triphenyl phosphate, TTBNPP = tris(tribromoneopentyl) phosphate, decaBDE = decabromodiphenyl ether, DP = bis(hexachlorocyclopentadieno)cyclooctane, TBBPA-BDBPE = tetrabromobisphenol A bis (2,3-dibromopropyl) ether, EBTEBPI = ethylene bis-tetrabromophthalimide, BTBPE = Bis(tribromophenoxy) ethane).

For the real substances data set, MCDA hazard scores (*f*) were calculated 15 times for each of the 17 substances, using different combinations of aggregation and curvature (weights were kept equal and constant). The objective hierarchy and the value function minima and maxima used were the same as in Zheng et al.^30^*f* was then used to rank the 17 substances, with the best performing alternative (i.e., highest median of *f*) being given a rank of 1 and the worst performing alternative being given a rank of 17. In Figure 4, the variability of the rank assigned to each substance as the method parameters were changed is shown by the bars. Thus, the bars reflect different MCDA ranks, not uncertainty of input data.

Figure 4 shows that, for most substances in this data set, the ranks assigned to each substance overlap with the ranks achieved by at least one other substance. This shows that the ranking also depends on the parameters chosen. A few substances consistently outperformed or underperformed relative to the other alternatives, regardless of the parameter combination applied. For example, melamine was consistently ranked as one of the best alternatives, whereas tetrabromobisphenol A bis (2,3-dibromopropyl) ether (TBBPA-BDBPE) was consistently ranked as one of the worst alternatives. However, the ranks of many of the other 15 substances strongly depend on the MCDA method parameters.

### Contrasting Results from GreenScreen and MCDA

Contrasting the results of the real substances data set between GreenScreen and MCDA (as shown in Figure 4) shows important differences. MCDA ranked melamine as the best performing alternative, while GreenScreen categorized all substances, including melamine, as Benchmark 1: “Avoid chemical of high concern.” This contrast highlights the limitations of the MCDA-MAVT analysis using this objective hierarchy and these value function minima and maxima. MCDA identified *f*_MA_ (the performance value of melamine) as a maximum, but this is a local, not a global maximum. There are substances outside this particular set of real substances that would have achieved *f* > *f*_MA_.

Contrasting the results of the hypothetical substances data set by GreenScreen and MCDA demonstrates the challenges associated with flexible method parameters. For example, the single combination of method parameters permitted by GreenScreen allows for a single outcome for Substance 64 (the hypothetical substance similar to some PFASs). In contrast, the 15 combinations of MCDA method parameters explored here allow for multiple outcomes for Substance 64. There are MCDA method parameter combinations where

*f*_substance64_ > *f*_substance86_
  e.g., *w*_P_ = low, curvature = linear, aggregation = additive
*f*_substance64_ = *f*_substance86_
  e.g., *w*_P_ = high, curvature = linear, aggregation = geometric-additive
*f*_substance64_ < *f*_substance86_
  e.g., *w*_P_ = equal, curvature = convex, aggregation = minimum

This range in MCDA outcomes demonstrates the necessity of standardized method parameters for the effective use of MCDA as a chemical alternative assessment method.

### Selecting MCDA Method Parameters to Align with REACH

**Figure 5:**
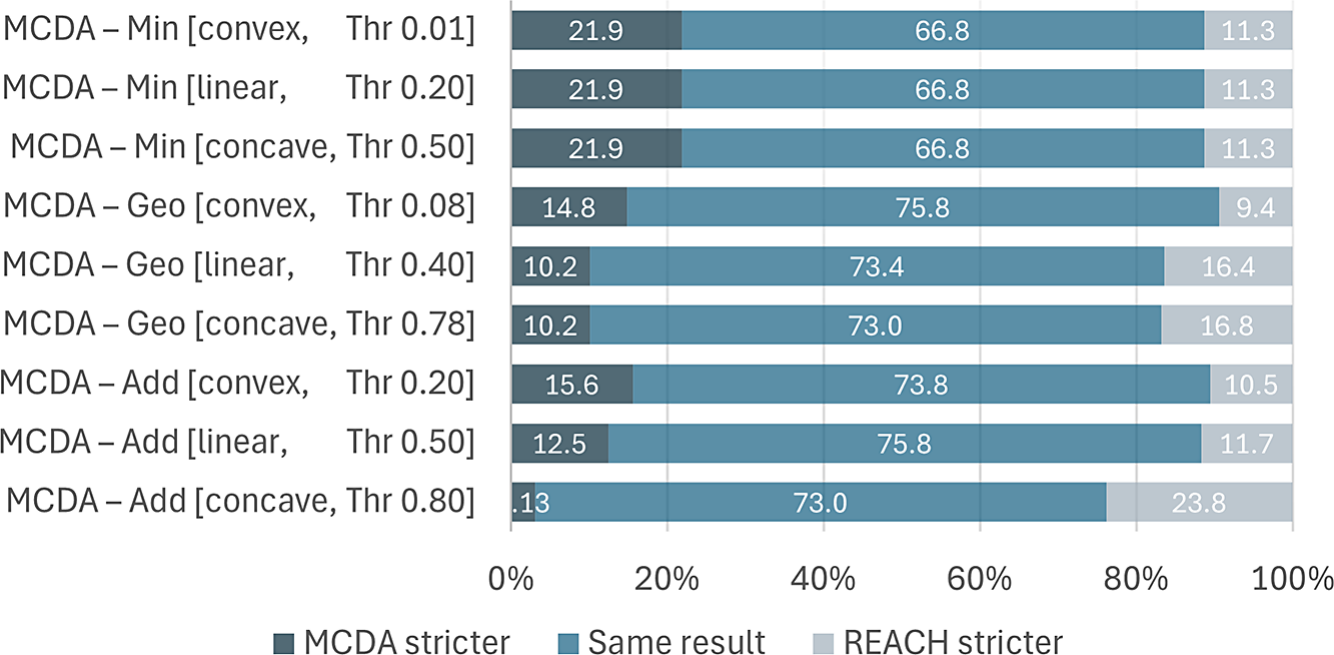
Percentage agreement for the hypothetical substances evaluated according to Article 57 of REACH and MCDA. For each substance, the results from REACH and MCDA could either be the same, or MCDA being stricter, or REACH being stricter. For MCDA, nine different method parameter combinations were investigated, with three different aggregations (Add = additive, geometric = Geo, or Min = minimum) and three different value functions (linear, exponential-concave and exponential-convex). Equal weights were used throughout (*w*_*i*_ = 0.25). Classification thresholds for the MCDA outcome, *f*, are given in the labels as “Thr.”

Aggregation and curvature were systematically tested with the hypothetical substances to see whether it is possible to align the MCDA outcome—using the relatively easy objective hierarchy applied in the previous publications—with the identification of potential SVHCs under REACH according to the criteria of Article 57. Figure 5 shows that the agreement between MCDA and REACH Article 57 was for all nine combinations between 67% and 76%.

None of the combinations of MCDA method parameters resulted in 100% agreement with REACH (i.e., same result = 100%). The combination that was previously used in other publications (additive aggregation with linear value function) resulted in 75.8% agreement. For 11.7% of the hypothetical substances, the evaluation with REACH Article 57 was stricter meaning that the MCDA evaluation would have overlooked regrettable substitutes. This shows that although MCDA is a very flexible method it is not possible to replicate Article 57 of REACH with the objective hierarchy given in Figure 2.

## Discussion

### Limitations of the Study

In terms of methods initially investigated, the present article is not comprehensive, there are other methods available, and it is possible that one of the unreviewed methods may outperform those reviewed in the present article.

In the assessment of GreenScreen, the decision tree for organic chemicals was used. There are other versions of this decision tree (e.g., for inorganic chemicals), but these were excluded from this study. Within this decision tree, only hazards that were relevant to Article 57 of REACH were considered, i.e., hazards that were considered within the GreenScreen decision tree, but not among the criteria for SVHCs, were excluded (e.g., physical hazards, such as explosiveness).

The MCDA methods used were limited to the MAVT approach; other MCDA methods such as ÉLECTRE were not considered. Additionally, the present article focuses on one stage of the MCDA process, namely how to structure the problem. Other aspects central to the practical application of MCDA, such as gathering hazard data, dealing with data gaps, or accurately quantifying the preferences of different stakeholders, were not considered.

### General Evaluation of the Hazard Assessment Methods

In this study, we assessed six different hazard assessment methods to determine their suitability for evaluating chemical alternatives to PFASs in the European marketplace under REACH. Two methods, GreenScreen and MCDA, were selected for detailed investigation. It is worth noting that the five methods excluded from further investigation may well be applicable in different scenarios, such as different regulatory contexts or other specific market requirements (e.g., aiming for market leadership vs fulfilling the legal requirements).

In the evaluation of the hypothetical substances, GreenScreen deviated from the criteria in Article 57 of REACH for 9% of the substances. Specifically, it indicated higher hazard for combinations of PT_eco_ and BT_eco_ than REACH, while signaling lower hazard for PBT substances. In addition, mobility was not included in the scope of GreenScreen, leading to an underestimation of the hazard of certain PFASs such as HFPO–DA.

The effect of varying MCDA method parameters was investigated in detail. It was found that substances with high hazard variability, such as PFASs, were particularly sensitive to changes of the MCDA parameter setting. This highlights the need for a careful selection of MCDA parameters in the assessment of chemical alternatives. The importance of MCDA parameter selection was highlighted further when the outcomes of GreenScreen and MCDA were compared. The real-substances data set illustrated that without fixed hazard thresholds, the MCDA outcome may not identify hazardous substances.

Within this study, attempts to find a combination of MCDA method parameters that aligned with the criteria of Article 57 of REACH were unsuccessful (see Figure 5). This may be because REACH relies on related objectives (e.g., PBT and vPvB both include persistence), while MCDA typically uses nonredundant objectives (e.g., see Figure 2). It may be possible to modify the objective hierarchy of MCDA so that the results align at the end with the criteria of Article 57 of REACH. The development and discussion of such an MCDA method is the subject of our accompanying paper London et al.^50^

### Using GreenScreen in Hazard Assessment

Many smaller companies with limited resources and technical expertise may use GreenScreen as it is user-friendly and relatively easy to implement. However, users should be aware of the fact that GreenScreen does not fully reflect the criteria of Article 57 of REACH, and of the method’s limited scope, excluding hazards such as mobility, GWP, and ODP. Finally, it is advisable to reevaluate whether the priorities, as set by the current version of the GreenScreen decision tree, still align with recent scientific developments. For example, in the current GreenScreen decision tree, the hazard of persistence alone is evaluated as Benchmark 3, indicating ‘Use but with room for improvement.’ However, there is increasing support for considering the hazard of persistence alone as being sufficient for regulation.^51^

### Using MCDA in Hazard Assessment

Researchers, larger organizations and governments may use MCDA for the hazard assessment part in AoA frameworks. With MCDA it is possible to choose parameter settings, and thus set priorities, which is in contrast to GreenScreen where parameters are set by the method’s authors. However, care should be taken in the parameter selection as it is not possible to mimic the criteria laid down in Article 57 of REACH with MCDA with the relatively simple objective hierarchy that has been proposed in previous publications. An MCDA method with a modified objective hierarchy is presented in our accompanying paper.^50^ We recommend using this method in the future for the hazard assessment under REACH.

## Supporting Information Available

Detailed descriptions of the investigated methods, published thresholds that can be used for the data normalization, and further details on the investigations into Greenscreen and MCDA (PDF)
Evaluation of the two methods, MCDA and Greenscreen, using the hypothetical substances (XLSX)
