# Supplementary material for: Are Hazard Assessment Methods in the Assessment of Chemical Alternatives Suitable for REACH?
Source: Environ Sci Technol. 2024 Oct 9;58(42):18811–21. doi: 10.1021/acs.est.4c03979 (PMC11500412; doi:10.1021/acs.est.4c03979)
Supplement: Supplementary file 1 — es4c03979_si_001.pdf [file es4c03979_si_001.pdf]

Supporting Information-1 to "Are Hazard Assessment Methods in the Assessment of Chemical Alternatives Suitable for REACH?"

Rachel L. London, Juliane Glüge,* and Martin Scheringer

Institute of Biogeochemistry and Pollutant Dynamics, ETH Zürich, 8092 Zürich, Switzerland

E-mail: juliane.gluege@usys.ethz.ch

Summary: 30 pages, 7 tables, 3 figures

Contents

S1 Overview of methods **S3**

S1.1 Investigations into selected methods S3

S1.1.1 Cradle to Cradle® S3

S1.1.2 GreenScreen® S5

S1.1.3 Multi Criteria Decision Analysis S7

S1.1.4 The GHS column model 2020 from IFA (Institute for Occupational Safety and Health of the German Social Accident Insurance) S7

S1.1.5 MA TURIs “Pollution Prevention Options Analysis System (P2OSys)” S8

S1.1.6 U.S. EPA Safer Choice Standard and Criteria S9

S1.2 Extended table: Overview of assessment of alternatives methods investigated S11

S1.3 Comparison of thresholds S15

S1.3.1 Persistence S15

S1.3.2 Bioaccumulation S18

S2 Investigations into GreenScreen® **S21**

S2.1 Assumptions made for the real substances dataset S21

S3 Investigations into MCDA **S24**

S3.1 Value functions equations S24

S3.2 Impact of varying value function on outcome for hypothetical substance datasetS24

S3.3 Investigations into the variability of MCDA – constant aggregation S26

References **S27**

S1 Overview of methods

S1.1 Investigations into selected methods

In the first part of the publication, assessment of chemical alternatives methods were identified from previous literature, compared, and it was investigated whether these methods use similar criteria as Article 57 of the regulation on Registration, Evaluation, Authorisation and Restriction of Chemicals (REACH). It was also assessed how per- and polyfluoroalkyl substances (PFAS) are treated. Each method is discussed here in more detail.

S1.1.1 Cradle to Cradle®

The Cradle to Cradle® method[1] is applied by businesses or consultants seeking to demonstrate the sustainability of their product to consumers. The final certification of the product (platinum, gold, silver, or bronze) communicates clearly and simply to the consumer a product's relative sustainability. A broad scope of sustainability categories is considered, falling into the five categories of "social fairness", "material reutilisation", "material health", "water stewardship", and "renewable energy and carbon management". Chemical hazard endpoints are considered within the broader category of "material health". Each of the chemical hazard endpoints is evaluated for each of the substances found within the product. The hazard endpoints within the method's scope include eleven human health endpoints (carcinogenicity, endocrine disruption, mutagenicity, reproductive and developmental toxicity, oral toxicity, dermal toxicity, inhalation toxicity, neurotoxicity, skin, eye, and respiratory corrosion / irritation, sensitization of skin and airways, and other), eight environmental health endpoints (fish toxicity, daphnia toxicity, algae toxicity, terrestrial toxicity, persistence, bioaccumulation, climatic relevance , and other), and two chemical class endpoints (organohalogens, toxic metals).

In 2022, the Cradle to Cradle® guidance was revised to align more closely with REACH,

in particular Article 57 (e), which specifies the hazard combination vPvB (very persistent and very bioaccumulative) as sufficient for restriction or authorisation in the EU,[2] and the hazard thresholds used to categorize persistence and bioaccumulation to align with the corresponding guidance.[3] This was done firstly by introducing a combined hazard flag ”persistence and bioaccumulation”, which allows the vPvB REACH category to be mimicked. The hazard categorization thresholds applied to the individual persistence and bioaccumulation endpoints have also been extended to include an additional hazard rating, PURPLE, which goes beyond RED as the most severe hazard rating. Secondly, the categorization thresholds used to assign a hazard rating for persistence (see Table S3) and bioaccumulation (see Table S6) have been modified to align with REACH (see Table S2 and S5 respectively). For example, for bioaccumulation, the hazard threshold for BAF (bioaccumulation factor) or BCF (bioconcentration factor) has been increased, with RED ($2000 < BCF/BAF \leq 5000$) now corresponding to the REACH threshold for ”bioaccumulative” and PURPLE ($BCF/BAF > 5000$) corresponding to the REACH threshold for ”very bioaccumulative”.

Cradle to Cradle® implicitly removes the need to determine the weights of different chemical endpoints, as a minimum aggregation method is used. The use of a minimum aggregation means that the overall classification of a chemical cannot exceed its worst endpoint classification (but the method allows for the presence of moderately classified substances to be mitigated using a precautionary exposure assessment). This means that substances which have a single severe hazard classification are considered unacceptable. This is stricter than Article 57 of REACH where specific endpoints are not sufficient for regulation unless found in combination with other specific endpoints. For example, under Article 57 of REACH, persistence alone is not sufficient for regulation. A substance must be persistent in combination with another hazard, such as bioaccumulation, to be regulated. However, the aim of Cradle to Cradle® is to identify and certify consumer products with exceptionally low environmental impact, therefore it is intended that the method is more precautionary than

the legal minimum.

Additionally, the organohalogen chemical class endpoint mandates that any chemical compound with a carbon-halogen bond is unacceptable. This, along with the minimum aggregation, means any PFAS evaluated using this method would be considered unacceptable. As a conclusion, one could say that this method is very precautionary, but it is not conclusive if all alternatives have a least one significantly hazardous endpoint.

The Cradle to Cradle® method has some limited flexibility in scope due to the inclusion of the two endpoints called "Other (Human Health)" and "Other (Environmental Health)". These two endpoints can be used to include additional hazards, that are not currently included in the standard Cradle to Cradle® assessment. For example, the assessment scope could be extended to include mobility, by including it within the assessment of the endpoint "Other (Environmental Health)".

S1.1.2 GreenScreen®

GreenScreen® is intended for use by businesses seeking to understand and reduce their use of hazardous chemicals. Using a decision tree, GreenScreen® categorizes a chemical alternative into one of four chemical benchmarks (1 = Avoid – chemical of high concern; 2 = Use but search for safer substitutes; 3 = Use but still opportunity for improvement; 4 = Prefer – Safer chemical). To determine the benchmark, the assessor proceeds through a decision tree using all available data for a chemical (Figure S1). Each node on the decision tree details a combination of hazard severity for the endpoints of persistence, bioaccumulation, human health toxicity, environmental toxicity, and physical hazard. The decision tree considers not only individual hazards, but also combinations such as PBT (persistent, bioaccumulative, and toxic) or vPvB (very persistent and very bioaccumulative). The hazard severity for each of these endpoints is determined by comparing all available data to thresholds, such as those found in Table S4 (persistence) and Table S7 (bioaccumulation). GreenScreen®

also specifies the minimum data requirements to determine the hazard severity for these endpoints and when a data gap should be assigned. There are some hazard classifications missing from the GreenScreen® method, such as mobility, global warming potential (GWP) or ozone depletion potential (ODP). Another drawback is that the assignment of the hazard combinations in the benchmarks of GreenScreen® is fixed without the flexibility to adapt. This makes GreenScreen® a very rigid method.

Please note, whilst the GreenScreen® Hazard Assessment Guidance (2018)[4] used within this paper does not specifically address PFAS or Organohalogens, GreenScreen® certification standards for seven product classes do include PFAS on their Restricted Substance lists. Substances on the Restricted substance list cannot be found in products above a certain concentration (typically 100ppm) for a product to receive a GreenScreen® Certification. The seven product classes with GreenScreen® certification standards including PFAS are: Furniture & Fabrics,[5] Cleaners & Degreasers in Manufacturing,[6] Single-Use Food Service Ware & Thermal Paper,[7] Reusable Food Packaging, Food Service Ware, & Cookware,[8] Firefighting Foam,[9] Medical Supplies & Devices,[10] and Textile Chemicals.[11]

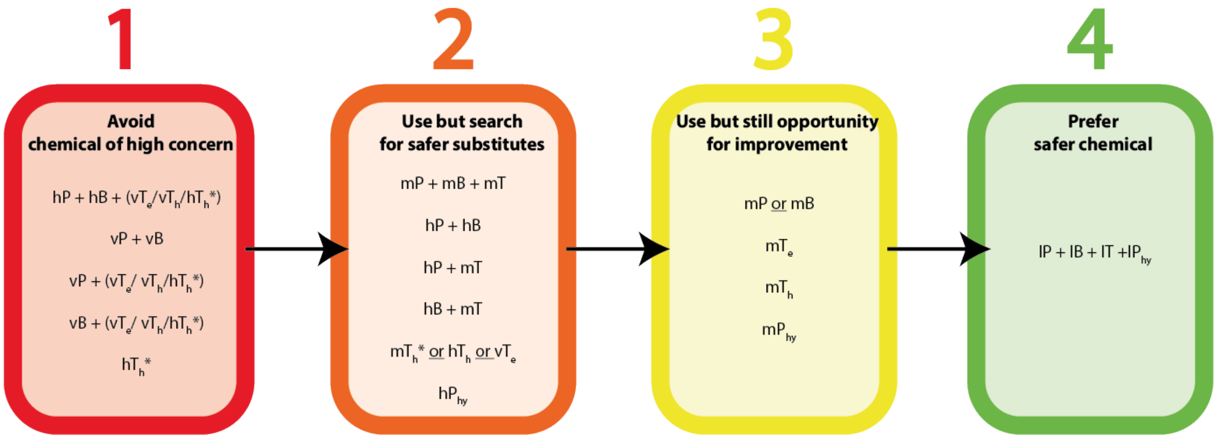

Figure S1: GreenScreen® decision tree, l = low, m = medium, h = high, v = very high, P = persistence, B = bioaccumulation, Phy = physical hazard, T_e = ecotoxicity, T_h = human toxicity, T_h* = a method defined subset of human toxicity endpoints

S1.1.3 Multi Criteria Decision Analysis

Multi Criteria Decision Analysis (MCDA) is a method used by decision makers to determine the best option amongst a set of alternatives. Originally developed in the field of economics, MCDA has been used by European institutes for policy decisions and is included as a recommended method in the technical guidance document for socio economic analysis in REACH restrictions.[12] Whilst there have been a couple of academic examples of their application in assessment of chemical alternatives[13–15] there have been few applications of the method by industry or regulators in the field of assessment of chemical alternatives.

Each MCDA is tailored to the problem being addressed and the alternatives available. First, the objectives of the policy or project are defined, and attributes that can be used to measure the success of these objectives, are identified. These attributes can be any measurable quality that can be used to distinguish between the alternatives available. For example, if one of the objectives for a desirable chemical alternative is low persistence, the half-life of the substance in different media could be used as a measurable attribute to distinguish between considered alternatives.

The decision method used by MCDA is that of a weighted sum. Three parameters can be varied to tailor the decision method to reflect the priorities of the decision makers – an objective's weight, the curvature of an attribute's value function, and the aggregation method. An MCDA should have objectives that are independent and without redundancy.[16] However, these requirements mean that it is not possible to replicate the criteria of Article 57 of REACH which can be combinations of hazards (e.g., PBT or PB – these objectives are neither completely independent of one another nor without redundancy).

S1.1.4 The GHS column model 2020 from IFA (Institute for Occupational Safety and Health of the German Social Accident Insurance)

The column method relies on data gathered from safety data sheets (SDS) and the Globally Harmonised System (GHS) classifications alone. It uses the GHS or SDS associated with

the product and assess the risk from negligible, low, medium, high, or very high. The method relies on the product as a whole, it does not assess individual components within that product. There are six hazard areas where a risk is assessed. The different alternatives are then compared. If there is a clearly less risky alternative available, it is advised to switch to that one. In the situation where there are alternatives which may be of lower risk in some categories but higher risk in others, the guidance advises that the decision maker weight each of the attributes based on what is most appropriate in their particular situation.

Persistence and bioaccumulation are not treated as individual hazard endpoints. However, substances that are PBT or vP vB would be a very high risk in environmental hazards column.[17]

Endpoints are limited to those currently covered by GHS. There is no flexibility to adapt the endpoints covered, to include endpoints such as endocrine disruption or mobility. In the situation where there is no clear alternative, no guidance is given on how to aggregate the performance in the different attributes/columns, this is left up to the discretion of the decision maker.

S1.1.5 MA TURIs "Pollution Prevention Options Analysis System (P2OSys)"

The P2OSys method[18] also relies on data gathered from SDS and GHS classifications alone. The SDS data are entered into an online tool that aggregates the data using a weighted sum. There are eight hazard categories (each with multiple subcategories): acute human effects (9), chronic human effects (7), ecological hazards (3), environmental fate and transport (3), atmospheric hazard (4), physical properties (9), process factors (10), and life cycle factors (6). Based on the hazard information entered, the tool assigns a value to each category between 2, good, and 10, bad. If there is no data for a subcategory, the tool removes that category from the calculation. Once a value has been assigned for all possible subcategories, an average is taken. The user can change the weighting if there is a particular category that they would like to place emphasis on.

Categories are combined in a weighted sum using additive aggregation. This means that this method allows poor performance in one category or subcategory to be compensated for by good performance in another category. For example, this method would fail to identify HFPO-DA as a problematic substance, as the poor performance in persistence, could be compensated for by good performance in acute toxicity. This is a problem if one is trying to replicate the criteria in Article 57 of REACH where poor performance in specific hazard combinations is sufficient for classification.

S1.1.6 U.S. EPA Safer Choice Standard and Criteria

The US EPA's Safer Choice Program Master Criteria for Safer Ingredients[19] is used for two US EPA programs - design for environment (which focuses on antibacterial products) and the safer choice standard (which focuses on a broader range of consumer products). These criteria determine whether a substance, and the products it is in, can be certified as a "safer choice". The criteria give hazard thresholds for ten endpoints: acute mammalian toxicity, carcinogenicity, genetic toxicity, neurotoxicity, repeated dose toxicity, respiratory sensitisation, skin sensitisation, environmental toxicity and fate, and eutrophication. All ten endpoints must be found to be below the hazard thresholds, for a substance to be considered a "safer choice". As there is no ability to compensate poor performance in one hazard with good performance in another hazard, this method can be classified as having a minimum aggregation method.

As with REACH Article 57, in the US EPA's Safer choice standard, the environmental endpoints of persistence, bioaccumulation, and toxicity are combined to categorize a substance. In both frameworks, single hazard thresholds are set for each environmental endpoint, which a substance either passes or fails. However, these methods differ in two aspects, the values at which the hazard thresholds are set, and how these environmental endpoints are combined. The hazard thresholds used by the US EPA are variable and stricter than those of REACH. For example, the bioaccumulation threshold for the US EPA standard is BCF or

BAF > 1000, stricter than that of REACH which is a BCF or > 2000 (persistent) or > 5000 (very persistent). The threshold for persistence is also variable, with the choice of 3 possible thresholds (a biodegradation half-life of 10 days, 28 days, or 60 days) being dependant upon the severity of the aquatic toxicity.

There are also differences in how each of the methods treats specific combinations of environmental endpoints. In REACH, a substance can be used unless it exceeds the thresholds for specific combinations of environmental endpoints, for persistence and bioaccumulation and toxicity (PBT), or very persistent and very bioaccumulative (vPvB). In addition to these combinations, the US EPA safer choice standard would also prevent substances being considered a "safer choice" if they were persistent and ecotoxic (PT), or persistent (P) alone. This difference is significant for this paper, as it would prevent many PFAS from being considered a "safer choice".

In addition to this, in 2023 an update to the EPA's safer choice and design for the environment standard was proposed. One of the proposed additions is a statement explicitly prohibiting packaging with this certification from including any intentionally added PFAS.[20]

S1.2 Extended table: Overview of assessment of alternatives methods investigated

Table S1: Extended overview of the investigated assessment of alternatives methods

**Hazard assessment method**	**Scope**	**Endpoints considered**	**Aggregation method**	**Weighting method**	**Flexibility**
**Cradle to cradle®**	Identify and certify consumer products with exceptionally low environmental impact	Eleven human-health endpoints (carcinogenicity, endocrine disruption, mutagenicity, reproductive & developmental toxicity, oral toxicity, dermal toxicity, inhalation toxicity, neurotoxicity, skin, eye, and respiratory corrosion / irritation, sensitization of skin and airways, and other), eight environmental endpoints (fish toxicity, daphnia toxicity, algae toxicity, terrestrial toxicity, persistence, bioaccumulation, climatic relevance, and other) and two chemical class endpoints (organohalogens, toxic metals)	Minimum aggregation within the individual categories as well as between the categories; some mitigation is possible using a precautionary exposure assessment	No weighting required	Little flexibility – some method elements (weighting, aggregation) are predefined. Additional endpoints (e.g. mobility) can be added using the environmental endpoint "other". Mitigation via the exposure assessment in certain cases gives some flexibility.

Table S1: Extended overview of the investigated assessment of alternatives methods

**Hazard assessment method**	**Scope**	**Endpoints considered**	**Aggregation method**	**Weighting method**	**Flexibility**
**GreenScreen®**	Used by businesses seeking to understand and reduce their use of hazardous chemicals.	Endpoints contributing to the categorization decision tree: persistence, bioaccumulation, physical hazard (flammability, and reactivity), ecotoxicity (acute aquatic toxicity and chronic aquatic toxicity), human toxicity (carcinogenicity, mutagenicity/genotoxicity, reproductive toxicity, developmental toxicity (incl. developmental neurotoxicity), endocrine activity, acute mammalian toxicity, systemic toxicity/organ effects-single exposure, neurotoxicity-single exposure, eye irritation, skin irritation, systemic toxicity/organ effects-repeated exposure, neurotoxicity-repeated exposure, respiratory sensitization, and skin sensitization)	Decision tree that considers not only individual hazards, but also combinations such as PBT or vPvB	No explicit weighting, but the decision tree contains implicit weighting	No flexibility

Table S1: Extended overview of the investigated assessment of alternatives methods

Hazard assessment method	Scope	Endpoints considered	Aggregation method	Weighting method	Flexibility
**Multi Criteria Decision Analysis (MCDA)**	Used by decision makers to determine the best option amongst a set of alternatives.	Not predefined	Not predefined, options include minimum, geometric, additive, geometric-additive, or maximum aggregation.	Not essential and depends on aggregation method used. If weighting is used, it is entirely flexible and determined by the decision maker.	Very flexible
**The GHS column model 2020 from IFA**	Used by small businesses and end users of chemical products. Very simple basic assessment. Assesses the chemical hazard of a complete product.	Six hazard areas (also called ”columns”): acute health hazards (single exposure), chronic health hazards (repeated exposure), environmental hazards, physico-chemical effects (fire, explosion, corrosion et al.), hazards from release behaviour, and process-related hazards	No guidance is given on how to aggregate the different endpoints/columns; this is left up to the discretion of the decision maker	In main method – implicit weighting, not possible to modify. Should no clear alternative be identified from main method - decision makers can decide what attributes are most important in their situation.	No flexibility – endpoints are limited to those currently covered by the GHS; there is no flexibility to include additional endpoints. In the case of an equal outcome, decision maker can decide relative importance of endpoints.

Table S1: Extended overview of the investigated assessment of alternatives methods

Hazard assessment method	Scope	Endpoints considered	Aggregation method	Weighting method	Flexibility
**MA TURIs "Pollution Prevention Options Analysis System (P2OSys)"**	Used by companies seeking to organise environmental, health and safety data about alternative chemicals, formulations, and processes.	8 categories (each with multiple subcategories): acute human effects (9), chronic human effects (7), ecological hazards (3), environmental fate and transport (3), atmospheric hazard (4), physical properties (9), process factors (10), and life cycle factors (6)	Weighted average	Typically, equal weighting between categories with data. Although there is the possibility to alter weightings if required	Limited flexibility – scope and aggregation fixed, but weight can be modified. Possible to exclude categories if desired.
**U.S. EPA Safer Choice Standard and Criteria**	Used by formulators of consumer goods seeking to be certified as a "safer choice".	10 attributes of concern: acute mammalian toxicity, carcinogenicity, genetic toxicity, neurotoxicity, repeated dose toxicity, respiratory sensitisation, skin sensitisation, environmental toxicity and fate, and eutrophication.	Minimum aggregation	No weighting required	No flexibility – certification method - fixed, scope, aggregation, implicit weighting

S1.3 Comparison of thresholds

S1.3.1 Persistence

Table S2: **Persistence thresholds:** REACH regulation Annex XIII, Chapter R.11: PBT/vPvB assessment - Table R.11-1

**Method**	**Classification [days]**	
	**Very persistent**	**Persistent**
**Half-life (marine water)**	60	60
**Half-life (fresh or estuarine water)**	60	40
**Half-life (marine sediment)**	180	180
**Half-life (fresh or estuarine water sediment)**	180	120
**Half-life (soil)**	180	120

Table S3: **Persistence thresholds:** Cradle to cradle's ® – material health assessment

Method	Classification				
	**Purple**	**Red**	**Yellow**	**Green**	**Grey**
**Half-life (water - fresh or estuarine) (days)**[a]	$t_{1/2}>60$	$40 \leq t_{1/2} \leq 60$	$16 \leq t_{1/2} \leq 40$	$t_{1/2} <16$	
**Half-life (water - marine) (days)**[a]			$16 \leq t_{1/2} \leq 60$		
**Half-life (soil or sediment from fresh or estuarine water) (days)**[a]	$t_{1/2} >180$	$120 \leq t_{1/2} \leq 180$	$16 \leq t_{1/2} \leq 120$		no data or inorganic substance
**Half-life (sediment from marine water) (days)**[a]			$16 \leq t_{1/2} \leq 180$		
**Half-life (air) (days)**[a]		$t_{1/2} > 2$		$t_{1/2} <2$	
**OECD 301 - Dissolved Organic Carbon (DOC) (% DOC removal)** [b]		<20%	20% <%DOC <70%	≥ 70% i.e. "readily biodegradable"	
**OECD 301 – Theoretical Oxygen Demand (ThOD) (% ThOD removal)** [b]			20% <%DOC <60%	≥ 60% i.e. "readily biodegradable"	
**OECD 302 or 304A - DOC removal**		<70% DOC removal	≥ 70% DOC removal		
**QSAR – BIOWIN**		recalcitrant	degradable within weeks to months	predicted to be readily biodegradable	

[a] The Cradle to Cradle® guidance gives this additional advice on half-life, and recommends EPISuite fugacity modelling to determine this:

> "The half-life value chosen to determine the final rating for this hazard endpoint must reflect the dominant environmental compartment in order to be meaningful"

[b] The Cradle to Cradle® guidance gives this additional advice on ThOD and DOC removal:

> "For a GREEN classification, either 70% removal of DOC or 60% removal of ThOD must be reached in a 10-day window within the 28-day time frame. The 10-day window begins once 10% biodegradation has been reached by DOC, ThOD, or ThCO2. If the 10% biodegradation is reached but the chemical in question does not reach the required degradation within 10 days, a YELLOW rating is given. In cases where 10% biodegradation does not trigger the 10-day window, a hazard of RED is given".

Table S4: **Persistence thresholds:** GreenScreen's® Chemical Hazard Criteria

**Method**	**Classification**					
	**very high (vH)**	**high (H)**	**moderate (M)**	**low (L)**	**very low (vL)**	**data gap (DG)**
**Half-life (soil or sediment)**	>180 days or recalcitrant	>60 to 180 days	16 to 60 days	<16 days or GHS "rapid degradability"	meets 10-day window in "ready biodegradation test"	
**Half-life (water)**	>60 days or recalcitrant	>40 to 60 days	16 to 40 days	<16 days or GHS "rapid degradability"	meets 10-day window in "ready biodegradation test"	
**Half-life (air)**	>5 days or recalcitrant	>2 to 5 days		<2 days		
**Long-range environmental transport**		evidence	suggestive evidence			
**Listed: EC – CEPA DSL [a]**	persistent					

[a] Canadian Categorization Decisions for Substances on the Domestic Substance List

S1.3.2 Bioaccumulation

Table S5: **Bioaccumulation thresholds:** REACH regulation Annex XIII, Chapter R.11: PBT/vPvB assessment - Table R.11-1

**Method**	**Classification**	
	**very bioaccumulative**	**bioaccumulative**
**Bioconcentration factor (in aquatic species)**	5000	3000

Table S6: **Bioaccumulation thresholds:** Cradle to cradle's ® – material health assessment

**Method**	**Classification**				
	**Purple**	**Red**	**Yellow**	**Green**	**Grey**
**BCF or BAF**[a]	>5000	2000 <BCF/BAF ≤ 5000	500 ≤ BCF/ BAF ≤ 2000	<500	no relevant data for classification
**log K_{OW}**				log K_{OW} <2	log K_{OW} >2 and no additional information
**Molecular weight**				>1000 g/mol	no relevant data for classification

[a] The Cradle to Cradle® guidance gives this additional advice on bioconcentration factors (BCF) and bioaccumulation factor (BAF):

> "QSAR estimated BCF may only be used when log K_{OW} is < 6 because the relationship is no longer linear above 6. When log K_{OW} is > 6, a measured/experimental BCF value is required. Alternatively, a QSAR estimated BAF may be used for log K_{OW} 6–8."

Table S7: **Bioaccumulation thresholds:** GreenScreen's® Chemical Hazard Criteria

**Method**	**Classification**					
	**very high (vH)**	**high (H)**	**moderate (M)**	**low (L)**	**very low (vL)**	**data gap (DG)**
**Bioaccumulation factor**	>5000	>1000 to 5000	>500 to 1000	>100 to 500	≤ 100	
**Bioconcentration factor**	>5000	>1000 to 5000	>500 to 1000	>100 to 500	≤ 100	
**log K_{OW}**	>5.0	>4.5 to 5.0	>4.0 to 4.5		≤ 4	
**Monitoring data (presence in humans or wildlife)**		evidence	suggestive evidence			
**Listed: EC – CEPA DSL[a]**	bioaccumulative					

[a] Canadian Categorization Decisions for Substances on the Domestic Substance List

S2 Investigations into GreenScreen®

S2.1 Assumptions made for the real substances dataset

The real substances dataset did not contain sufficient data to perform a GreenScreen® assessment in line with the GreenScreen® guidelines. So, to allow the investigation of the GreenScreen® method using the real substances dataset, a number of assumptions were made. The assumptions related to data quality, additional data not being considered, data type, data granularity, and a precautious approach being taken upon countering any ambiguity.

Data quantity. In the real substances dataset, the data quantity for some hazard endpoints was fewer than required for a GreenScreen® evaluation. Rather than assign a data gap, if any data were available for an endpoint this was considered sufficient for classification. For example, for the endpoint of endocrine disruption, GreenScreen® requires data for five different pathways. For a substance to be considered of low risk, negative data is required for: androgenicity, anti-androgenicity, estrogenicity, anti-estrogenicity, and thyroid effects. However, the real substances dataset only provided data for androgen binding, estrogen binding, and thyroid binding. Therefore, according to GreenScreen® guidelines, insufficient data on endocrine disruption is present to classify the substances. Nonetheless, for this investigation, substance classification proceeded based on the available data.

Additional data not considered. The GreenScreen® guidelines state that all available reliable data for each hazard endpoint should be gathered prior to the assessment. However, the only data considered were the data provided in the real substances dataset, potentially leading to imprecise or erroneous conclusions.

For example, for melamine, the data in the real substances dataset led to a low hazard rating for the developmental toxicity endpoint of this substance. However, the REACH registra-

tion for melamine, gives the substance a GHS Reproductive toxicity classification of Repr.2 (H361f: Suspected of damaging fertility).[21]

Data type. GreenScreen® guidance specifies the type of data required to determine a hazard for each endpoint. Using the real substances dataset, the data types available were not sufficient to reach classifications for several endpoints. Therefore, QSAR results were assumed to be equivalent to certain GHS classifications. For example, for the carcinogenicity endpoint, the real substances dataset provided QSAR results indicating chemical structures' carcinogenic potential on a continuous scale from 1.0 (worst) to 3.0 (best). These were converted to approximate GHS classifications:

- 1.0 = Category 1 Carcinogen – Known or presumed human carcinogen
- 2.0 = Category 2 Carcinogen – Suspected human carcinogen
- 3.0 = Not carcinogenic

Data granularity. When converting between data types (e.g., from QSAR to GHS), a disparity in the granularity of the data lead to ambiguity and inaccuracy in the result. For example, with the endpoint of eye irritation, GHS has four categories:

- Category 1 (Causes serious eye damage)
- Category 2A (Irritant)
- Category 2B (Mild Irritant)
- No classification

However, the real substances dataset provided QSAR results evaluating the hazard of eye irritation as a binary option of either 1 (worst) or 3 (best). These QSAR results were converted to GHS as:

- 1 = Category 1 (Causes serious eye damage)
- 3 = No classification

Precaution in response to ambiguity. Where a classification for an endpoint was ambiguous, a more precautionary approach was favoured, and the most severe classification was assigned. For example, in the real substances dataset, the endpoint of carcinogenicity for the substance of melamine was given a value of 1.66 (this is an average of three QSAR models from different VEGA QSAR models for carcinogenicity). This value of 1.66 fell between the two values of 1.0 (i.e., GHS Category 1 Carcinogen) and 2.0 (i.e., GHS Category 2 Carcinogen). The value was rounded down to the nearest whole number and melamine was classified as a GHS Category 1 Carcinogen.

S3 Investigations into MCDA

S3.1 Value functions equations

This paper used the linear (Equation S1) and exponential (Equation S2) value functions as defined in Haag et al.[22] for the ValueDecisions app.

$$\text{Linear value function} \quad \nu\left(x\right) = z = \left(\frac{x - x_{\text{worst}}}{x_{\text{best}} - x_{\text{worst}}}\right) \tag{S1}$$

$$\text{Exponential value function} \quad \nu\left(x, c\right) = \frac{1 - \exp\left(-c \cdot z\right)}{1 - \exp\left(-c\right)} \quad \text{for } c \neq 0 \tag{S2}$$

Where x is the hazard score of each attribute. The degree to which the value function is curved is set by c, which in this paper is exponential-concave $c = 5$ and exponential–convex $c = -5$.

S3.2 Impact of varying value function on outcome for hypothetical substance dataset

It is to be expected that changing the value function will change the outcome for all substances considered within the hypothetical substance dataset, regardless of the variation of hazard severity of that substance. By varying the curvature, Figure 3 of the main paper explores the impact of changing the value function used to normalise a hazard score x to a value ν that can then be aggregated to determine a score for the alternative f. The three value functions explored in this paper (linear, exponential-convex, and exponential-concave, see Eq. S1 and S2) converge at the best possible hazard score x_{best} and the worst possible hazard score x_{worst}. For the hypothetical substance dataset, these were defined as $x_{\text{best}} = 1.0$ and $x_{\text{worst}} = 0.0$. For all other values of x between x_{best} and x_{worst}, the three value functions diverge. This is shown in Figure S2.

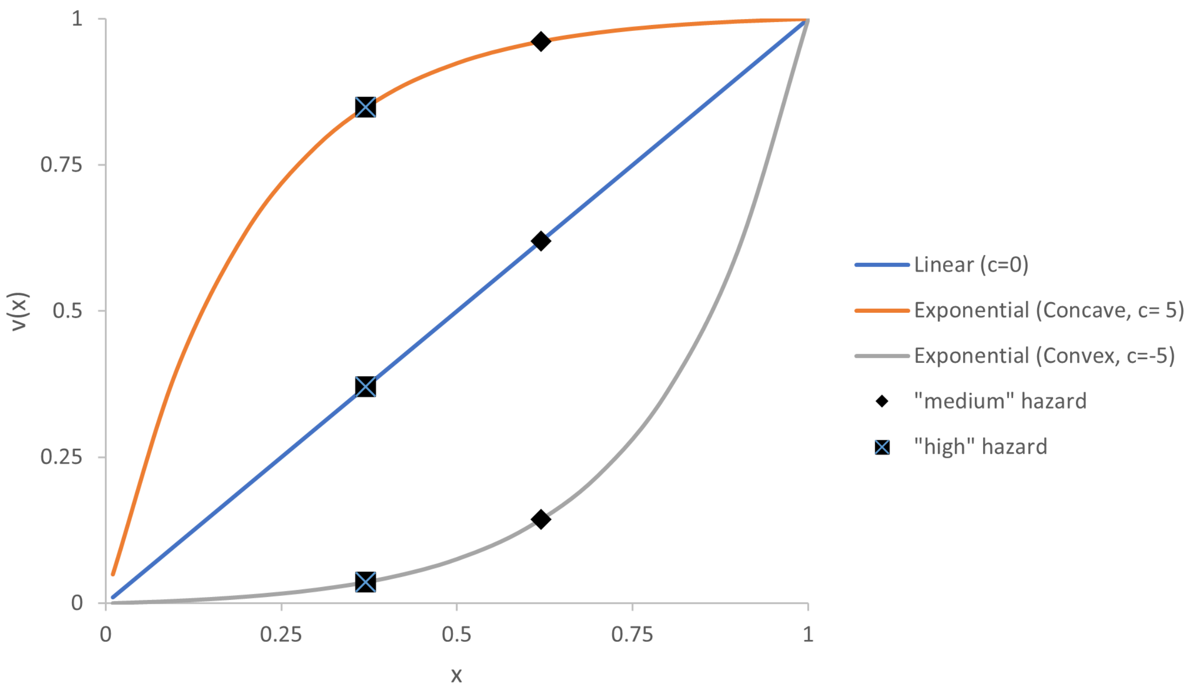

Figure S2: Graph showing the conversion of hazard score x to hazard value $\nu(x)$ using different value functions (linear, exponential – concave, and exponential – convex)

As can be seen in Figure S2:

- The linear function has a constant derivation for all values of x.
- The concave exponential function has a large derivation close to x_{worst}, and a small derivation close to x_{best}.
- The convex exponential function has a small derivation close to x_{worst}, and a large derivation close to x_{best}.

The practical implication of this is:

- when x_{best} or x_{worst}:
  - $\nu(x)$ will be the same for all three value functions
- in all other cases:
  - $\nu(x)$ will be different for all three value functions

S3.3 Investigations into the variability of MCDA – constant aggregation

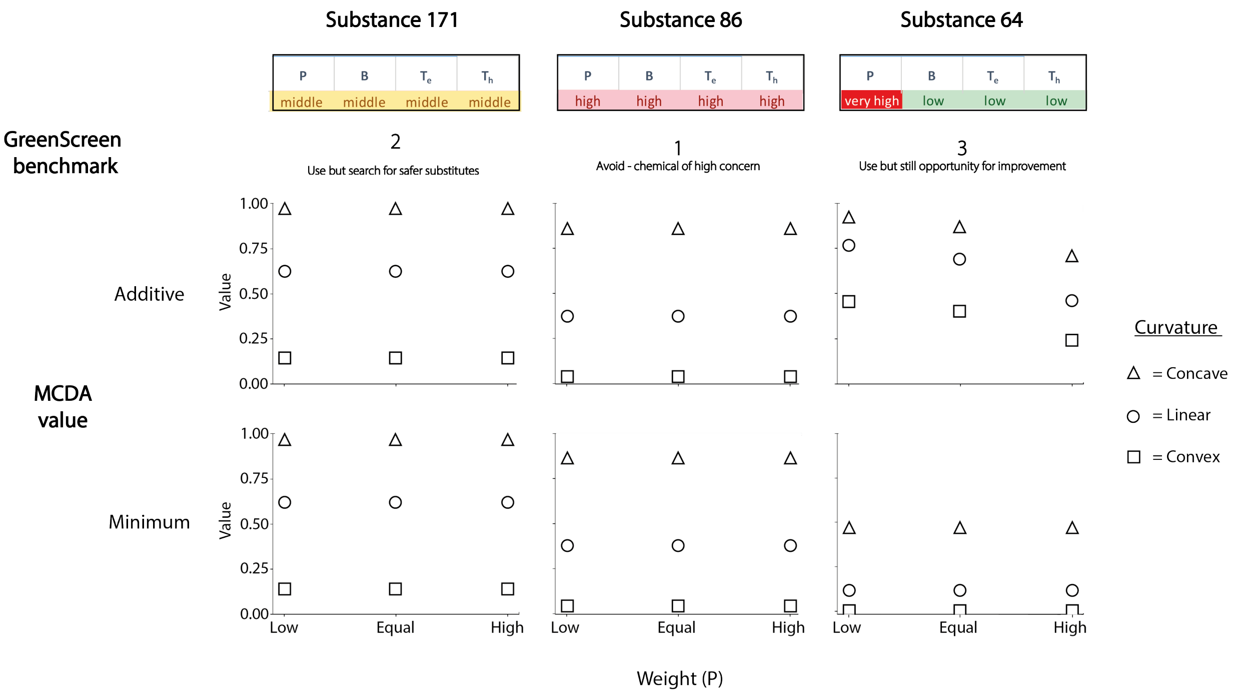

Figure S3: Variability of MCDA – Values for three of the hypothetical substances (2 x low variation of hazard severity, 1 x high variation of hazard severity). Value as a function of curvature and weight, aggregation kept constant (top row – additive aggregation, bottom row – minimum aggregation)

References

(1) Cradle to Cradle Products Innovation Institute Material health assessment methodology. 2022; https://cdn.c2ccertified.org/resources/certification/standard/MTD_Material_Health_Assessment_FINAL_021522.pdf, (accessed 2024-02-14).

(2) European Parliament and Council Regulation (EC) No 1907/2006 of the European Parliament and of the Council of 18 December 2006 concerning the Registration, Evaluation, Authorization and Restriction of Chemicals (REACH). Official Journal of the European Union, 2006; https://eur-lex.europa.eu/legal-content/EN/TXT/?uri=uriserv:OJ.L_.2006.396.01.0001.01.ENG, (accessed 2024-06-01).

(3) European Chemicals Agency *Guidance on Information Requirements and Chemical Safety Assessment Chapter R.11: PBT/vPvB assessment - version 4.0*; 2023; pp 1–205.

(4) Clean Production Action GreenScreen® for Safer Chemicals - Hazard Assessment Guidance. 2018; https://www.greenscreenchemicals.org/static/ee_images/uploads/resources/GreenScreen_Guidance_v1_4_2018_01_Final.pdf, (accessed 2024-07-25).

(5) Clean Production Action Greenscreen Certified - Standard for Furniture & Fabrics. 2023; https://www.greenscreenchemicals.org/images/ee_images/uploads/resources/CPA_GSC_Furniture_Fabric_Standard_v1-2(1e).pdf, (accessed 2024-06-11).

(6) Clean Production Action Greenscreen Certified - Standard for Cleaners & Degreasers in Manufacturing. 2021; https://www.greenscreenchemicals.org/images/ee_images/uploads/resources/CPA_GSC_Cleaners_Degreasers_Standard_v1-0.pdf, (accessed 2024-06-11).

(7) Clean Production Action Standard for Single-Use Food Service Ware & Thermal Paper. 2023; https://www.greenscreenchemicals.org/images/ee_images/uploads/

resources/CPA_GSC_Single_Use_Food_Ware_Standard_v1-2.pdf, (accessed 2024-06-11).

(8) Clean Production Action Greenscreen Certified - Standard for Reusable Food Packaging, Food Service Ware, & Cookware. 2024; https://www.greenscreenchemicals.org/images/ee_images/uploads/resources/CPA_GSC_Reusables_Standard_v1-0-1.pdf, (accessed 2024-06-11).

(9) Clean Production Action Greenscreen Certified - Standard for Standard for Firefighting Foam. 2023; https://www.greenscreenchemicals.org/images/ee_images/uploads/resources/GSC-Firefighting-Foam-Standard_1.pdf, (accessed 2024-06-11).

(10) Clean Production Action Greenscreen Certified - Standard for Medical Supplies & Devices. 2022; https://www.greenscreenchemicals.org/images/ee_images/uploads/resources/CPA-GSC-Medical_v1-1.pdf, (accessed 2024-06-11).

(11) Clean Production Action Greenscreen Certified - Standard for Textile Chemicals. 2022; https://www.greenscreenchemicals.org/images/ee_images/uploads/resources/GSC_Textile_Chemicals.pdf, (accessed 2024-06-11).

(12) European Chemicals Agency Guidance on Socio-Economic Analysis – Restrictions. 2008; https://echa.europa.eu/documents/10162/2324906/sea_restrictions_en.pdf/2d7c8e06-b5dd-40fc-b646-3467b5082a9d, (accessed 2024-07-26).

(13) Malloy, T. F.; Sinsheimer, P. J.; Blake, A.; Linkov, I. Use of multi-criteria decision analysis in regulatory alternatives analysis: A case study of lead free solder. *Integr. Environ. Assess. Manag.* **2013**, *9*, 652–664.

(14) Zheng, Z.; Peters, G. M.; Arp, H. P. H.; Andersson, P. L. Combining in Silico Tools with Multicriteria Analysis for Alternatives Assessment of Hazardous Chemicals: A

Case Study of Decabromodiphenyl Ether Alternatives. *Environ. Sci. Technol.* **2019**, *53*, 6341–6351.

(15) Zheng, Z.; Arp, H. P. H.; Peters, G.; Andersson, P. L. Combining In Silico Tools with Multicriteria Analysis for Alternatives Assessment of Hazardous Chemicals: Accounting for the Transformation Products of decaBDE and Its Alternatives. *Environ. Sci. Technol.* **2021**, *55*, 1088–1098.

(16) European Chemicals Agency Guidance on the preparation of socio-economic analysis as part of an application for authorisation. 2011; https://echa.europa.eu/documents/10162/2324906/sea_authorisation_en.pdf/aadf96ec-fbfa-4bc7-9740-a3f6ceb68e6e, (accessed 2024-07-26).

(17) Institut für Arbeitsschutz der Deutschen Gesetzlichen Unfallversicherung The GHS Column Model 2020. 2020; https://publikationen.dguv.de/widgets/pdf/download/article/3737, (accessed 2022-01-30).

(18) Massachusetts Toxics Use Reduction Institute P2OASYS Tool to Compare Materials Updated. 2021; https://www.turi.org/Our_Work/Alternatives_Assessment/Alternatives_Assessment/Tools_and_Methods/P2OASys_Tool_to_Compare_Materials/P2OASys_Tool_To_Compare_Materials_Updated, (accessed 2023-06-01).

(19) U.S. Environmental Protection Agency EPA's Safer Choice Program Master Criteria for Safer Ingredients. 2012; https://www.epa.gov/sites/default/files/2013-12/documents/dfe_master_criteria_safer_ingredients_v2_1.pdf, (accessed 2023-06-01).

(20) U.S. Environmental Protection Agency EPA's Safer Choice and Design for the Environment (DfE) Standard. 2023; https://downloads.regulations.gov/EPA-HQ-OPPT-2023-0520-0003/content.pdf, (accessed 2024-07-26).

(21) European Chemicals Agency REACH Registration Dossier: Isotridecanol, ethoxylated. 2023; https://echa.europa.eu/registration-dossier/-/registered-dossier/13803/1/2, (accessed 2023-10-12).

(22) Haag, F.; Aubert, A. H.; Lienert, J. ValueDecisions, a web app to support decisions with conflicting objectives, multiple stakeholders, and uncertainty. *Environ. Model. Softw.* **2022**, *150*, 105361, Eawag, Switzerland: www.eawag.ch/en/DA/ValueDecisions.
